# Prevalence, Spinal Alignment, and Mobility of Lumbar Spinal Stenosis with or without Chronic Low Back Pain: A Community-Dwelling Study

**DOI:** 10.1155/2011/340629

**Published:** 2011-05-05

**Authors:** Naohisa Miyakoshi, Michio Hongo, Yuji Kasukawa, Yoshinori Ishikawa, Yoichi Shimada

**Affiliations:** Department of Orthopedic Surgery, Akita University Graduate School of Medicine, 1-1-1 Hondo, Akita 010-8543, Japan

## Abstract

Although lumbar spinal stenosis (LSS) occurs almost universally with aging, little is known regarding its actual prevalence and relationships to chronic low back pain (CLBP) in the general population. The presence of CLBP in subjects with LSS may have negative impacts on spinal alignment and mobility. This study evaluated the prevalence of LSS using a self-administered, self-reported history questionnaire in 630 community-dwelling individuals ≥50 years old. Subjects with LSS were further divided into LSS+CLBP and LSS alone groups, and spinal alignment and mobility were compared using a computer-assisted device. Prevalence of LSS was 10.8% in this cohort. Subjects in the LSS+CLBP group (*n* = 46) showed a significantly more kyphotic lumbar spinal alignment with limited lumbar extension (*P* < .05), resulting in a stooped trunk compared to subjects in the LSS alone group (*n* = 22). However, no significant difference in spinal mobility was seen between groups.

## 1. Introduction

Low back pain (LBP) is a primarily cited condition among problems linked to postural imbalance [1, 2]. Recurrent or chronic LBP (CLBP) is estimated to occur in 35–79% of patients [3, 4]. Particularly in older individuals, CLBP is at least partially related to degenerative changes associated with aging [5]. The degenerative process (spondylosis) involves the intervertebral discs, facet joints, vertebral bodies, and spinal ligaments. These spondylotic changes often induce lumbar spinal stenosis (LSS).

LSS is a well-recognized spinal disorder that commonly affects older adults. The pain and disability associated with degenerative LSS represent a substantial and growing health problem among the elderly [6, 7]. Clinically symptomatic LSS occurs when compression of the nerve roots or cauda equina causes pain, numbness, and tingling, or weakness in the lower extremities. With progressive compression, ambulation may be severely affected (neurogenic claudication). Although LSS has been widely studied in the clinical setting, particularly for surgical cases, few population-based studies have been attempted [8]. In addition, the association between LSS and CLBP remains unclear.

Spinal alignment and mobility are important factors for spinal function. Loss of lumbar lordosis correlates well with the incidence of CLBP in adults [9, 10]. Patients with a less mobile spine may show more severe symptoms. However, to the best of our knowledge, no previous community-based studies have assessed spinal alignment and mobility in subjects with LSS with or without CLBP. The objectives of this study were thus (1) to determine the prevalence of LSS in a district of Japan and (2) to compare differences in spinal alignment and mobility between LSS subjects with CLBP and without CLBP, to evaluate the impact of CLBP on spinal alignment and mobility in subjects with LSS.

## 2. Materials and Methods

Recruitment activities were designed to enroll a cohort of healthy, ambulatory, community-dwelling individuals ≥50 years old who participated in a public health examination in Kamikoani, Akita, Japan. With a population of approximately 2900, the town of Kamikoani is in a mainly rural (farming/forestry) district and contains one of the highest percentages of elderly subjects among towns in Japan. In 2010, 45.6% of the population was ≥65 years old. Individuals who were institutionalized, unable to walk without the assistance of another person, or unable to provide self-reported data were excluded. A total of 630 eligible subjects in Kamikoani were enrolled in this study, representing approximately 22% of the total town. Subjects included 218 men and 412 women with a mean age of 71.4 years (range, 50–94 years).

All participants completed a self-administered, self-reported history questionnaire (SSHQ) for the diagnosis of LSS [11], as described later. If the participants were diagnosed with LSS using the SSHQ, they were further asked whether they had clinically relevant CLBP, and body mass index (kg/m^2^), spinal inclination, and spinal alignment (angle of kyphosis) and range of motion (ROM) of the thoracic and lumbar spine were measured. These measured variables were compared between LSS subjects with CLBP (LSS+CLBP group) and those without CLBP (LSS alone group). 

This study was conducted in accordance with the Declaration of Helsinki. The study protocol was approved by the ethics committee of the government health authorities of Kamikoani. All subjects provided written informed consent prior to examination.

### 2.1. Diagnosis of LSS

Diagnosis of LSS was obtained using an SSHQ developed by Konno et al. [11]. This diagnostic tool consists of 10 key questions (Table 1). Sensitivity and specificity of the SSHQ were 84% and 78%, respectively [11]. The area under the receiver operating characteristic curve was 0.782 [11]. In test-retest analysis, the intraclass correlation coefficient (ICC) for first and second tests was 85% [11].

### 2.2. Definition of Clinically Relevant CLBP

LBP was considered clinically relevant if the participant answered that pain had been moderately to severely bothersome, or if the participant needed any medical treatment. CLBP was defined if the participant had clinically relevant LBP lasting >1 year [12, 13]. All other pain episodes were classified as not clinically relevant CLBP.

### 2.3. Measurement of Spinal Kyphosis Angles, ROMs, and Inclinations

Kyphosis angles and ROMs of the thoracic (T1-T12) and lumbar (L1-L5) spine were measured using a device for computerized measurement of surface curvature (SpinalMouse; Idiag, Volkerswill, Switzerland) in an upright position and at maximum flexion and extension [14]. Details regarding this device have been published previously [15]. The device consists of a mobile unit of 2 rolling wheels interfacing with a base station through telemetry. By sliding the mobile unit along the spinal curvature, sagittal spinal alignment is calculated and displayed on the computer monitor. Repeating this process with the patient in flexion and extension of the spine allows measurement of ROM [15]. Spinal inclination, defined as the angle between a line from the center of T1 to S1 and a perpendicular line [16], was also measured using SpinalMouse in an upright position and at maximum flexion/extension. SpinalMouse delivers consistently reliable values for standing curvatures and ROM [15, 17]. Post and Leferink [15] reported that interrater ICCs for curvature measurement with SpinalMouse were greater than 0.92. Mannion et al. [17] reported that the intrarater ICCs ranged from 0.82 to 0.83 and interrater ICCs ranged from 0.81 to 0.86. In addition, our previous studies have shown that thoracic and lumbar angles of kyphosis and spinal ROMs measured using the SpinalMouse correlated strongly with those measured on spinal radiography (*r* = 0.804, *r* = 0.863, and *r* = 0.783, resp.; *P* < .0001) [18].

### 2.4. Statistical Analysis

All data are presented as mean and standard deviation (SD). Statistical analysis was performed using StatView version 5.0 software (Abacus Concepts, Berkeley, CA). Statistical differences between the two groups were compared using an unpaired *t*-test. Values of *P* < .05 were considered statistically significant.

## 3. Results

### 3.1. Prevalence of LSS

**Figure 1:**
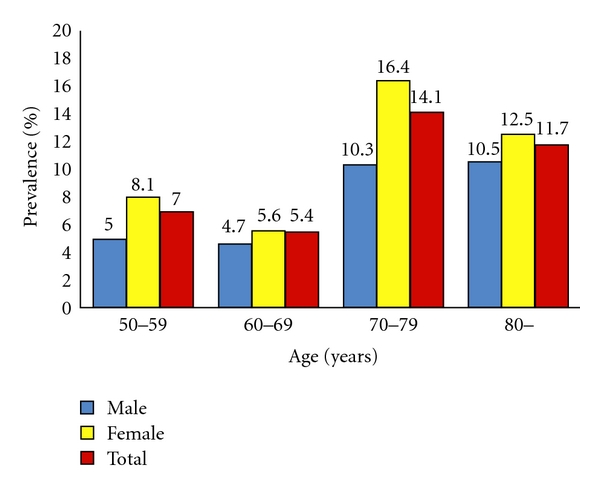
Prevalence of lumbar spinal stenosis with age.

Overall, 68 participants (10.8%) were diagnosed with LSS. These comprised 19 men and 49 women, with a mean age of 73.9 years (range 55–87 years). Prevalences of LSS in men and women were thus 8.7% and 11.9%, respectively. The prevalence of LSS tended to increase with age (Figure 1).

### 3.2. Prevalence of CLBP in Subjects with LSS

In 68 subjects with LSS, 46 subjects (67.6%) complained of clinically relevant CLBP. No significant differences were apparent between the LSS alone group (*n* = 22) and the LSS+CLBP group (*n* = 46) with regard to age, gender distribution, height, weight, or body mass index (Table 2). Subjects who had both LSS and CLBP were distributed across all ages (Table 3). Prevalence of CLBP in subjects with LSS seemed to be irregular with age, probably because the numbers of LSS subjects were smaller in younger age groups.

### 3.3. Comparisons of Spinal Alignment and Mobility in LSS Subjects with or without CLBP

With regard to spinal alignment, the LSS+CLBP group showed a significantly lower angle of thoracic kyphosis in an upright position, and significantly higher angles of lumbar kyphosis in upright, flexed, and extended positions compared to the LSS alone group (Table 4). The LSS+CLBP group also showed significantly higher angles of spinal inclination in the upright and extended positions compared to the LSS alone group. However, with regard to spinal mobility, no significant differences in any variables with spinal ROMs were seen between the LSS alone and LSS+CLBP groups (Table 5).

## 4. Discussion

Little is known about the actual prevalence of symptoms associated with LSS in the general population. The present study showed that the prevalence of LSS among individuals ≥50 years old in a healthy aged cohort from a rural district in Japan was 10.8%. This number is similar to that reported by Vogt et al. [8] in their population-based survey, which found that among 5995 men ≥65 years old who participated in the Osteoporotic Fractures in Men Study, 12.2% experienced symptoms of numbness/tingling/weakness extending into the leg, suggestive of LSS.

However, the prevalence of LSS may vary between studies, as the diagnosis of LSS remains problematic with different diagnostic criteria applied in different studies. Typically, the preliminary diagnosis is based on clinical symptoms and signs. After referral to an orthopedic surgeon or neurosurgeon, imaging studies confirm the diagnosis. In the present study, the diagnosis of LSS was obtained based on an SSHQ to identify LSS with a high sensitivity, specificity, and reproducibility [11]. This self-reported diagnostic tool is useful for a population-based survey. This diagnostic tool was developed according to a retrospective derivation study, a prospective derivation study, and a validation study using more than 500 surgical cases diagnosed by board-certified spine surgeons approved by the Board of the Japanese Society for Spine Surgery and Related Research as the gold standard for diagnosing LSS [11]. 

The clinical hallmark finding of LSS is neurogenic intermittent claudication, presenting as intermittent pain or paresthesia in the legs brought on by walking and standing, and classically relieved with flexion. However, other symptoms are also frequent. Amundsen et al. reported that the most common symptoms in patients with LSS were back pain including LBP (prevalence, 95%), claudication (91%), leg pain (71%), weakness (33%), and voiding disturbances (12%) [19, 20]. LBP is thus considered one of the major symptoms of LSS.

However, relationships between LSS and CLBP remain unclear. In particular, the exact sagittal profile of the spine and spinal mobility in subjects with LSS alone or in combination with CLBP has still yet to be exactly defined. The present study showed that 67.6% of subjects with LSS experienced CLBP and that spinal alignment differed between subjects with LSS and CLBP and subjects with LSS alone, irrespective of the equivalent age and gender distributions. In this study, spinal alignment and mobility were measured using a computer-assisted device that is useful for fieldwork. The results of the present study indicate that subjects with LSS and CLBP showed more kyphotic lumbar spinal alignment with a stooped trunk (i.e., increased spinal inclination) compared to subjects with LSS alone. Thoracic kyphosis was decreased in subjects with LSS and CLBP, probably because of compensation for increased lumbar kyphosis due to CLBP. In addition, lumbar flexion was not restricted by the presence of CLBP in subjects with LSS, whereas lumbar extension was limited. However, total spinal mobility was not restricted by the presence of CLBP in subjects with LSS.

Associations of increased lumbar kyphosis and LBP among non-LSS subjects have been discussed in the literature [1, 21–23]. Jackson and McManus [1] found a significant decrease in lumbar lordosis (i.e., increased lumbar kyphosis) for the LBP group compared with normal controls. Results from several other studies support the concept that increased lumbar kyphosis is associated with increased LBP [21–23]. Increased spinal kyphosis is known to have significant associations with decreased back extensor strength [24]. Thus, although we did not measure back extensor strength in this study, decreased back extensor strength might be a causal factor for CLBP in the LSS+CLBP group.

The limitations of this study should be noted. First, neither radiographic nor magnetic resonance imaging (MRI) data were available in this study to evaluate spondylotic severity and/or neurologic compression. In addition to lumbar kyphosis, several other factors including disc degeneration, facet osteoarthritis, and neurologic compression might differ between individuals with and without CLBP. Furthermore, the prevalence of lumbar instability, spondylolysis, and spondylolisthesis was unknown in this cohort. Second, this study did not evaluate pain intensity. Since differences in pain intensity may be associated with different modifications of spinal curves, a wide variety of physical and mental outcome scores for LBP including the Oswestry Disability Index, Roland Morris Disability Questionnaire, and the simple Visual Analogue Scale may help to address this problem. However, because this study was performed as part of a public health examination, conducting radiographic or MRI examination was impossible, and insufficient time was available to obtain these validated questionnaires and scales for LBP.

## 5. Conclusions

In conclusion, the prevalence of LSS among individuals ≥50 years old in a rural Japanese cohort was 10.8%. Among subjects with LSS, 67.6% had CLBP. Subjects with LSS and CLBP showed more kyphotic lumbar spinal alignment with limited lumbar extension, resulting in a stooped trunk compared to subjects with LSS alone. However, the presence of CLBP did not affect total spinal mobility in subjects with LSS.

## Figures and Tables

**Table 1:** Key questions from a self-administered, self-reported history questionnaire for the diagnosis of lumbar spinal stenosis.

Q1:	Numbness and/or pain is present in the thighs down to the calves and shins
Q2:	Numbness and/or pain increases in intensity after walking for a while, but relieved by taking a rest
Q3:	Standing for a while brings on numbness and/or pain in the thighs down to the calves and shins
Q4:	Numbness and/or pain is reduced by bending forward
Q5:	Numbness is present in both legs
Q6:	Numbness is present in the soles of both feet
Q7:	Numbness arises around the buttocks
Q8:	Numbness is present, but pain is absent
Q9:	A burning sensation arises around the buttocks
Q10:	Walking nearly causes urination

Each question is assigned one point. Lumbar spinal canal stenosis is diagnosed if (1) the score was 4 points from Q1 to Q4 or (2) the score was more than 1 point from Q1 to Q4 and more than 2 points from Q5 to Q10.

**Table 2:** Demographic characteristics in LSS subjects with or without CLBP.

Variables	LSS alone (*n* = 22)	LSS+CLBP (*n* = 46)	Total (*n* = 68)
Male : female	8 : 14	11 : 35	19 : 49
Age (years)	72.8 (6.0)	74.4 (7.1)	73.9 (6.7)
Height (cm)	151.6 (7.3)	148.5 (9.7)	150.8 (9.0)
Weight (kg)	55.0 (8.5)	52.6 (8.8)	54.4 (9.2)
Body mass index (kg/m^2^)	24.1 (4.3)	23.9 (3.0)	24.0 (3.4)

LSS: lumbar spinal stenosis; CLBP: chronic low back pain.

Data are presented as mean (SD).

**Table 3:** Prevalence of CLBP in subjects with LSS with age.

	Age (years)				Total
	50–59	60–69	70–79	80–	
Male					
No. of LSS	1	2	12	4	19
No. of LSS+CLBP	1	1	7	2	11
Prevalence of CLBP in LSS (%)	100	50	58.3	50	57.9

Female					
No. of LSS	3	7	32	7	49
No. of LSS+CLBP	2	5	21	7	35
Prevalence of CLBP in LSS (%)	66.6	71.4	65.6	100	71.4

Total					
No. of LSS	4	9	44	11	68
No. of LSS+CLBP	3	6	28	9	46
Prevalence of CLBP in LSS (%)	75	66.6	63.7	81.8	67.6

LSS: lumbar spinal stenosis; CLBP: chronic low back pain.

**Table 4:** Comparisons of spinal alignment in LSS subjects with or without CLBP.

Variables	LSS alone (*n* = 22)	LSS+CLBP (*n* = 46)	Total (*n* = 68)
Upr thoracic kyphosis angle (°)	40.7 (5.3)	35.0 (11.9)*	36.9 (10.5)
Upr lumbar kyphosis angle (°)	−10.7 (12.4)	0.1 (20.4)*	−3.6 (18.7)
Upr spinal inclination (°)	5.5 (6.8)	12.1 (11.9)*	9.8 (10.9)
Flex thoracic kyphosis angle (°)	45.2 (10.1)	40.3 (13.1)	41.9 (12.3)
Flex lumbar kyphosis angle (°)	9.5 (17.0)	24.9 (18.6)*	19.6 (19.4)
Flex spinal inclination (°)	85.9 (30.2)	87.1 (25.3)	86.7 (26.8)
Ext thoracic kyphosis angle (°)	32.7 (10.4)	26.7 (15.0)	28.8 (13.8)
Ext lumbar kyphosis angle (°)	−18.5 (9.9)	−5.7 (20.7)*	−10.0 (18.7)
Ext spinal inclination (°)	−14.8 (11.9)	−5.2 (18.3)*	−8.4 (17.0)

*Significant difference versus LSS alone group (*P* < .05 by unpaired *t*-test).

LSS: lumbar spinal stenosis; CLBP: chronic low back pain; upr: upright position; flex: flexed position; ext: extended position.

Data are presented as mean (SD).

**Table 5:** Comparisons of spinal ROM in LSS subjects with or without CLBP.

Variables	LSS alone (*n* = 22)	LSS+CLBP (*n* = 46)	Total (*n* = 68)
Thoracic flex-upr ROM (°)	7.9 (4.7)	7.1 (5.9)	7.4 (5.5)
Thoracic upr-ext ROM (°)	9.1 (5.8)	10.8 (9.5)	10.2 (8.4)
Thoracic ROM (°)	17.0 (7.6)	18.0 (10.7)	17.7 (9.7)
Lumbar flex-upr ROM (°)	20.1 (12.6)	25.2 (14.4)	23.5 (13.9)
Lumbar upr-ext ROM (°)	10.7 (9.9)	8.2 (6.8)	9.1 (8.0)
Lumbar ROM (°)	30.9 (16.1)	33.4 (16.0)	32.5 (16.0)
Total flex-upr ROM (°)	28.0 (14.5)	32.3 (16.1)	30.9 (15.6)
Total upr-ext ROM (°)	19.9 (10.8)	19.0 (11.9)	19.3 (11.4)
Total ROM (°)	47.9 (18.6)	51.4 (21.6)	50.2 (20.5)

LSS: lumbar spinal stenosis; CLBP: chronic low back pain; upr: upright position; flex: flexed position; ext: extended position; ROM: range of motion.

Data are presented as mean (SD).
